# Supplementary material for: The contributions of biological maturity and experience to fine motor development in adolescence
Source: Sci Rep. 2026 Jan 21;16:5917. doi: 10.1038/s41598-026-36220-y (PMC12894658; doi:10.1038/s41598-026-36220-y)
Supplement: Supplementary file 1 — Supplementary Material 1 [file 41598_2026_36220_MOESM1_ESM.docx]

**Bone age correction method**

Bone age (BA) data in the present study was corrected based on the data in Utczás et al (2017) article. Although the mean BA values for the 6-16 year chronological age (CA) range averaged across all subjects are roughly equal to CA, the mean BA differs slightly from CA in some age ranges. It was present especially for boys, e.g. BA is slightly higher at 15-16 years and lower at 13 years of CA. A balanced BA measurement device is expected to show that the average BA is equal to CA in all age ranges.

To eliminate these differences, the BA values were corrected to minimize the deviation of the mean BA from the mean CA in each age group of one year length.

To achieve this goal, a correction table was created, which gives a correction value for each CA with a precision of 0.01 years, which is subtracted from the measured BA value. The table was also constructed separately for boys and girls, as there were gender differences.

To determine the values of the correction table, we first sorted the subjects in the database (by sex) in ascending order of CA.

We then created a table with the CA in the first column from age 6 to age 18.5 with a 0.01 step interval. For each CA, the mean BA was determined by finding all subjects within ±0.5 years of CA of the measured values and taking the mean of their BAs. This gave the mean BA with a resolution of 0.01 years.

The average BA values were then smoothed with a 1 Hz (1 Hz = 1 year) low-pass filter. The resulting values became the values of the correction table. The BA values of the subjects measured in the present study were then corrected using the correction table. To do this, the subject's CA was searched in the table and the corresponding correction value was subtracted from the subject's BA.

The steps to determine the correction table were done in Excel, except for the low-pass filtering, which was performed in MatLab.

Reference

Utczás, K., Muzsnai, A., Cameron, N., Zsakai, A., & Bodzsar, E. B. (2017). A comparison of skeletal maturity assessed by radiological and ultrasonic methods. American Journal of Human Biology, 29(4), e22966. https://doi.org/10.1002/ajhb.22966
